# IgG4-related disease of the paratestis in a patient with Wells syndrome: a case report

**DOI:** 10.1186/s13000-014-0225-5

**Published:** 2014-12-09

**Authors:** Takashi Karashima, Yoshinori Taniguchi, Tsutomu Shimamoto, Tomoya Nao, Hiroshi Nishikawa, Satoshi Fukata, Masayuki Kamada, Keiji Inoue, Kentaro Oko, Hideki Nakajima, Shigetoshi Sano, Manabu Matsumoto, Naoto Kuroda, Yoshihiro Kamei, Taro Shuin

**Affiliations:** Department of Urology, Kochi Medical School, Kohasu, Oko, Nankoku, Kochi 783-8505 Japan; Department of Endocrinology, Metabolism and Nephrology, Kochi Medical School, Nankoku, 783-8505 Japan; Department of Dermatology, Kochi Medical School, Nankoku, 783-8505 Japan; Laboratory of Diagnostic Pathology, Kochi Medical School Hospital, Nankoku, 783-8505 Japan; Department of Diagnostic Pathology, Kochi Red Cross Hospital, Kochi, 780-0062 Japan; Kamei Private Clinic, Kochi, 780-0085 Japan

**Keywords:** IgG4-related disease, Paratestis, Pseudotumor, Wells syndrome

## Abstract

**Background:**

We report a case of a 33-year-old man who presented with immunoglobulin (Ig)G4-related disease (IgG4-RD) forming a pseudotumor in the left paratesticular region during oral administration of corticosteroid for Wells syndrome, which involves cellulitis with eosinophilia.

**Case presentation:**

The patient was introduced to our institution from a private hospital with a 3-month history of asymptomatic left scrotal mass. A 5-cm diameter nodule was palpable in the left scrotum. Tumor lesion in the left paratestis involving the epididymis and spermatic cord was observed on computed tomography and magnetic resonance imaging. Blood testing showed no abnormalities other than a minimal increase in C-reactive protein levels. Urine examination likewise revealed no significant findings. Left radical orchidectomy was performed under a diagnosis of left paratesticular neoplasm suspected as malignant tumor. The tumor was pathologically identified as IgG4-RD of the left paratestis involving the epididymis and spermatic cord.

**Conclusions:**

We present a first description of IgG4-RD in a patient with Wells syndrome and the ninth case of IgG4-RD in a scrotal organ, and discuss this very rare entity with reference to the literature.

**Virtual Slides:**

The virtual slide(s) for this article can be found here: http://www.diagnosticpathology.diagnomx.eu/vs/13000_2014_225

## Background

Immunoglobulin (Ig)G4-related disease (IgG4-RD) is a recently defined, emerging clinical entity characterized by diffuse fibrosis or mass-forming pseudotumor with infiltration of IgG4-positive plasma cells [1]. IgG4-RD often produces a neoplastic entity and has been found in multiple locations throughout the body, including organs of the genitourinary system such as the kidney and prostate. However, only 8 cases of IgG4-RD in the scrotum have been reported to date [2-6]. We report the ninth case of scrotal pseudotumor associated with IgG4-RD and discuss the clinical and histopathological characteristics of this rare neoplastic entity.

Wells syndrome is a rare dermatosis, also known as eosinophilic cellulitis, characterized by recurrent inflammatory dermatosis with erythematous plaques [7]. We present the first description of IgG4-RD in a patient with Wells syndrome and also discuss the relationship between both of these hyper-immune diseases.

## Case presentation

**Figure 1:**
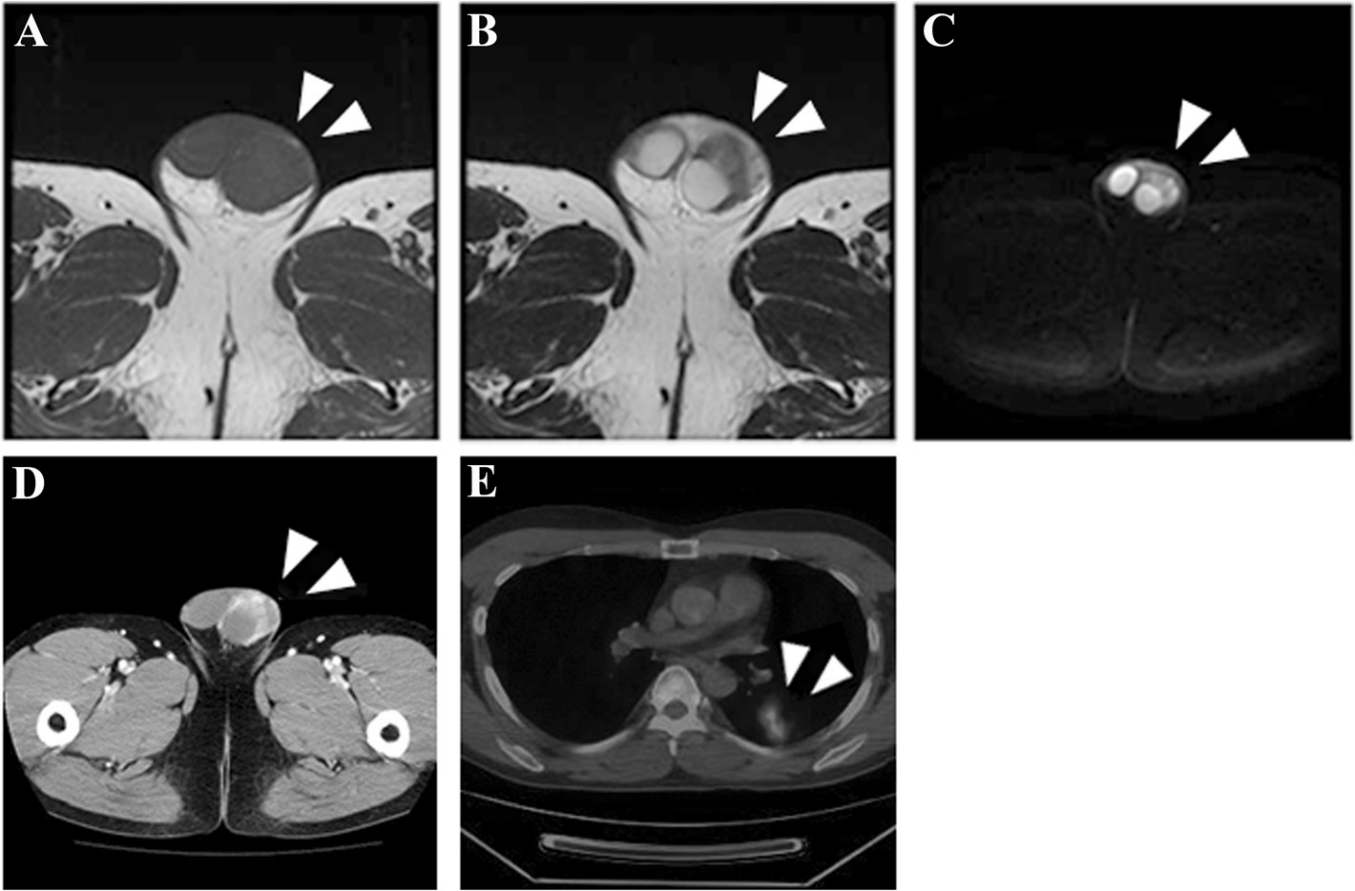
**Pre-operative diagnostic imaging.** Magnetic resonance imaging of the left scrotum shows a low-intensity mass in the paratesticular region on T1 **(A)** -and T2 **(B)** -weighted imaging, and areas of high signals on diffusion-weighted imaging (white arrows; **C**). *The paratesticular lesion is irregularly enhanced on CT imaging* **(D)** *. FDG-PET/CT imaging shows accumulation of FDG in left lung* **(E)**.

**Figure 2:**
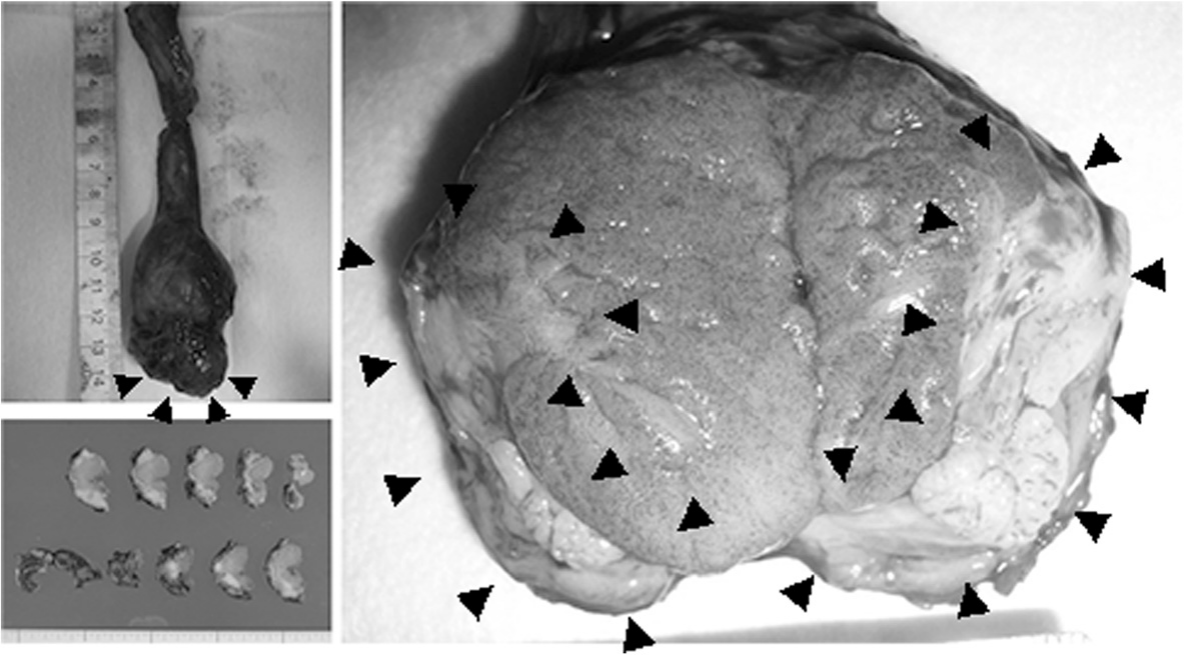
**Macroscopic findings of surgical specimen.** A mass is seen in the tail of the left epididymis, extending to the tunica vaginalis and parenchyma of the left testis (black arrows).

A 33-year-old man had noticed swelling with underlying palpable mass in the left scrotum 3 months earlier. The patient was introduced to Kochi Medical School from a private hospital on suspicion of testicular cancer. He had taking oral corticosteroid for hyper-eosinophilia diagnosed as Wells syndrome since he was 27 years old. He had undergone incisional drainage of a periproctal abscess at 30 years old. No history of exposure to tuberculosis was evident. Physical examination revealed a non-tender, indurated, solid mass at the lower pole of the left epididymis, possibly also involving the testis. Scrotal ultrasound demonstrated a solid, heterogeneous mass involving the left epididymis and extending into the testis. Levels of serum tumor markers including β-human chorionic gonadotropin, α-fetoprotein and lactate dehydrogenase were within normal limits. C-reactive protein level was slightly elevated, at 0.5 mg/dL (normal, <0.3 mg/dL). *Contrast-enhanced computed tomography (CT) of the left scrotum revealed a 31 × 28-mm diameter left epididymal mass showing irregular contrast and poorly defined margins.* Magnetic resonance imaging (MRI) of the left scrotum revealed a hypointense mass in the left epididymis on T1- and T2-weighted imaging. Part of the capsule of the left testis showing as a low-intensity layer was poorly marginated on T2 imaging. On diffusion-weighted imaging, the mass showed some high signals (Figure 1). Left radical orchidectomy was performed under a presumed diagnosis of left paratesticular tumor. The tumor was an elastic, hard, whitish nodule. The origin of the tumor was macroscopically speculated to be the left epididymis, and the part of tumor was unmargined the tunica albuginea and spermatic cord of the left testis (Figure 2).

**Figure 3:**
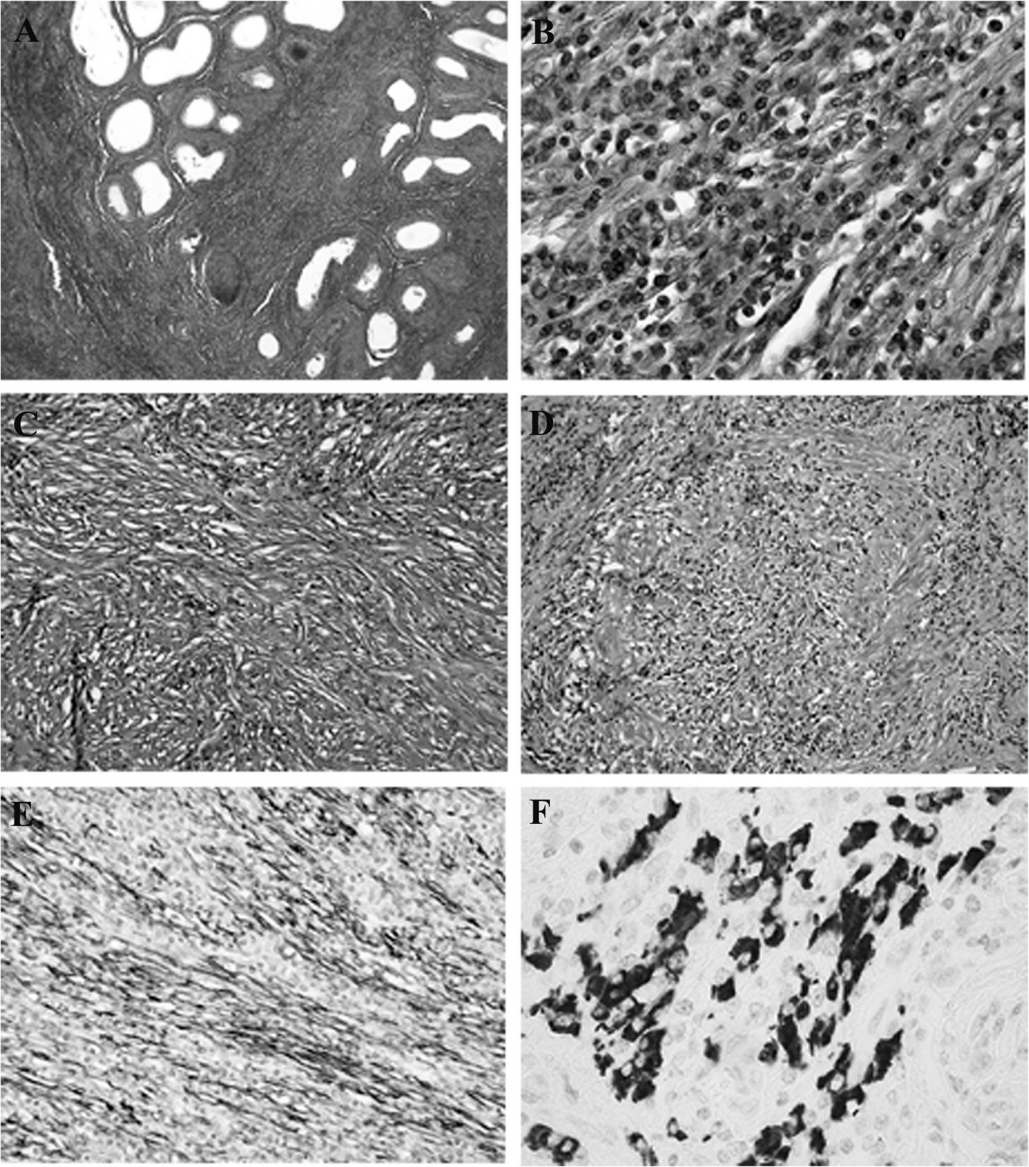
**Microscopic findings of surgical specimen.** Hematoxylin-eosin staining of the paratesticular mass reveals widespread fibrosis and lymphocyte aggregation from the left epididymis to the spermatic cord (*×40;* **A**). Spindle cell proliferation with chronic inflammatory cells mostly comprised plasma cells with neither atypia nor mitosis (*×200;* **B**). Myofibroblastic cells with marked fibrosis reveals a storiform pattern (*×100;* **C**). Obliterative phlebitis is apparent (*×100;* **D**). *For immunostaining, sections were stained with anti-alpha-smooth muscle actin (α-SMA) (1A4, 1:800, DAKO, Glostrup, Denmark) and -IgG4 (HP6025, 1:1280; ZYMED Laboratories, CA, USA) antibodies by using automated immunostainer (Ventana Benchmark, Tucson, AZ, USA). Spindle cells are positive for α-SMA, indicating myoepithelial cells* (×*100;* **E**)*. More than 10 IgG4-positive plasma cells/HPF are seen on anti-IgG4 immunostaining* (×*400;* **F**)*.*

Histopathology of the tumor demonstrated spindle-shaped epithelial and polygonal cell proliferations with storiform fibrosis. Little atypia and few mitoses were identified among spindle-shaped epithelial cells. Plasmacytes, lymphocytes and eosinophils had infiltrated into the tumor. Typical obstructive phlebitis was also observed. Positive immunostaining was obtained for vimentin, α-smooth muscle actin (αSMA) and desmin (focally), but negative results were seen for CD34, S100 protein, p53, anaplastic lymphoma kinase (ALK), cytokeratin (CAM5.2), calretinin, Wilms’ tumor-1, thrombomodulin, epithelial membrane antigen and lymphatic endothelial marker in the spindle cells, identifying the myofibroblastic cells. IgG4-positive cells comprised 50% among the cells staining positively for IgG, and the number of IgG4-positive plasma cells/high-power fields (HPF) were more than 10 (Figure 3). The histological finding of pseudotumor was consistent with IgG4-RD in the left epididymis.

The patient displayed accumulation of ^18^ F-fluorodeoxyglucose (FDG) in the left lung on FDG-positron emission tomography/CT (FDG-PET/CT) (Figure 1E) after the orchidectomy. The patient has since been continuously taking *1.5 mg of oral dexamethasone sodium phosphate equally to 10 mg of prednisolone and also to 37.5 mg of hydrocrtisone.* The left lung lesion had disappeared on follow-up FDG-PET/CT at 24 months postoperatively and no evidence of recurrence was seen at the site of resection.

We have received a consent from the patient for publication of the present report.

## Discussions

IgG4-RD is a common autoimmune disease in various organs, including the submandibular gland, lung, pancreas, kidney, retroperitoneum and prostate. IgG-RD often causes pseudotumor consisting of IgG4-positive plasma cell and intense fibrosis. Testis and paratestis with involvement of the epididymis and spermatic cord are rare regions for IgG4-RD. Nine cases, including this case, with scrotal lesions associated with IgG4-RD are summarized in Table 1. Median age was 33 years (range, 19–74 years). Peak age at onset might show a biphasic pattern, in adolescence and old age. Chief complaints were mostly palpable nodule in the scrotum. Multifocal lesions including in the pancreas, retroperitoneum, submandibular gland and prostate described in Cases 4, 5 and 8 were all in elderly patients at 73, 74 and 64 years old, respectively. IgG4-RD in adolescent patients may differ from that in the elderly. *Thirty three years old at the present might be in the elderly case, because he had multi-organ disease including lung lesion on FDG-PET/CT.* All patients underwent surgical excision with orchidectomy or local excision of nodules. Histopathological findings were myofibroblastic proliferation, infiltration of lymphocytes and plasma cells, and a high ratio of IgG4-positive cells.

**Table 1:** **Brief summary of cases reported in the literature of scrotal IgG4 related pseudotumor**

**No.**	**Reference**	**Era**	**Age**	**Presentation**	**Diagnosis and multiorgan disease**	**Treatment**	**Serum IgG4 value (mg/dL)**	**Histology**
1	Bösmüller et al. [2]	2011	23	Bilateral Palpable paratesticular multiple indolent floating masses on both sides	Paratesticular fibrous pseudotumor	Surgical excision of nodules	Not described	Plasma cell infiltration, IgG4/IgG ratio 44%
2	Bösmüller et al. [2]	2011	25	Right swelling of the right testis with pain	Paratesticular inflammatory pseudotumor	Semicastration.	Not described	Dense myofibroblastic proliferation with sparse mitoses, IgG4/IgG ratio 48%
3	Bösmüller et al. [2]	2011	52	Right palpable paratesticular nodules	Paratesticular fibrous pseudotumor	Surgical excision of nodules	Not described	Plasma cell infiltration, IgG4/IgG ratio 46%
4	Hart et al. [3]	2012	73	Painless right scrotal mass	Paratesticular pseudotumor, Autoimmune pancreatitis, Retroperitoneal fibrosis	Right inguinal radical orchiectomy	391	Lymphoplasmacytic infiltration, storiform pattern of fibrosis, IgG4/IgG ratio 60%
5	Migita et al. [4]	2012	74	Left paratesticular mass	Paratesticular pseudotumor, Submandibular gland inflammation, prostatitis	Left semicastration	505	Fibrosis with lymphocytic and plasmacytic infiltrations, IgG4/IgG ratio 85%
6	Dickmann et al. [5]	2013	19	Right painless intrascrotal mass	Spermatic cord inflammatory pseudotumor	Local excision	Not determined	Partially storiform, spindle-like cells and lymphofollicular infiltration, IgG4/IgG ratio 40%
7	Dickmann et al. [5]	2013	28	Right painless intrascrotal mass	Spermatic cord inflammatory pseudotumor	Local excision	Not determined	As above
8	de Buy Wenniger et al. [6]	2013	64	Bilateral scrotal pain	Testicular pseudotumor, Autoimmune pancreatitis, Retroperitoneal fibrosis	Bilateral orchidectomy	Not described	Plasma cell-rich infiltrate and myofibroblastic spindle cell proliferation around the seminiferous tubules, IgG4/IgG ratio 50% in right testis and 80% in left testis
9	Our case	2014	33	Left palpable scrotal mass	Paratesticular pseudotumor, Lung lesion	Radical orchidectomy	31.8	Spindle epithelial and polygonal cell proliferation with intense fibrous, plasmacyte, lymphocyte and eosinophil infiltration, IgG4/IgG ratio 50%

Comprehensive diagnostic criteria for IgG4-RD were described in 2011, setting the following 3 criteria: 1) clinical examination showing characteristic diffuse/localized swelling or masses in single or multiple organs; 2) hematological examination showing elevated serum IgG4 concentrations (>135 mg/dl); and 3) histopathological examination showing marked lymphocyte and plasmacyte infiltration and fibrosis, and infiltration of IgG4-positive plasma cells (ratio of IgG4-positive/IgG-positive cells >40% and >10 IgG4-positive plasma cells/HPF) [8]. The present case was identified as “probable”, meeting criteria 1 and 3. Serum IgG4 concentration in our patient was 31.8 mg/dl before surgery. The patient had receiving oral administration of corticosteroid, which might have decreased serum IgG4 concentrations to within normal limits. As the patient had a history of developing dyspnea and cyanosis due to upper airway edema when not taking corticosteroid, the patient has continued taking corticosteroid.

*Various differential diagnoses show inflammation-related tumors involving true or pseudo-neoplasia.* Inflammatory pseudotumor related to microbial infection, trauma or postoperative status must be diagnosed by excluding other possibilities. In the present case, histopathological findings of less mitotic myofibroblastic proliferation with storiform and swirling fibrosis, lymphoplasmacytic infiltration, obliterative phlebitis and an abundance of IgG4-positive cells met the criteria allowing final diagnosis of pseudotumor associated with IgG4-RD. Immunomarkers offered additional definitive and exclusive diagnosis of IgG4-RD. In the present case, the differential diagnosis should have included inflammatory myofibroblastic tumor (IMT), a typical neoplastic entity with positive immunostaining of ALK. Staining for vimentin and αSMA was diffusely positive and desmin was focally positive, but negative results were seen for CD34, S100 protein, p53 and ALK in the present case, indicating myoepithelial cell proliferation and excluding neoplasias such as IMT. However, consideration should be given to the fact that cases of scrotal IMT have been reported to often show negative immunostaining for ALK [9].

Wells syndrome is an uncommon inflammatory dermatosis, first described in 1971 by Wells. Clinical appearance is variable, combined with the histopathological presence of eosinophilic infiltrates and flame figures in the absence of vasculitis, and a relapsing remitting course is usually seen. The present case was diagnosed as Wells syndrome based on systemic cellulitis combined with histopathological presence of eosinophilic infiltration of the skin 6 years earlier. A case with hypereosinophilic syndrome was reported with overlapping IgG4-RD in 2012. The author implied that hypereosinophilic syndrome interacted with IgG4-RD [10]. In Wells syndrome, an eosinophilic infiltration mediated by T-helper cells 2 cytokines such as interleukin-5 and −13 is based on immune-pathogenesis in common with IgG4-RD [11,12]. Histopathological findings for the skin lesion showed features as eosinophilic infiltration common with the paratesticular lesion, *but not positive immunostaining with anti-IgG4 antibody in the present case. A common immune-etiology of Wells syndrome and IgG4-RD were still controversial.*

In the literature, not all patients could be diagnosed preoperatively because of the lack of characteristic findings for scrotal IgG4-RD. Our patient also underwent radical orchidectomy, even though CT, MRI and biological testing were performed. Orchidectomy should be avoided for pseudotumor associated with IgG4-RD and pharmacotherapy such as corticosteroid and immunosuppressive agents should be applied instead.

## Conclusions

The paratestis is a clinically rare region for IgG4-RD. We have reported a case of IgG4-RD of the paratestis in a patient treated with corticosteroid for Wells syndrome, a systemic eosinophilic disease.

## Consent

Written informed consent was obtained from the patient for publication of this Case report and any accompanying images. A copy of the written consent is available for review by the Editor of this journal.

## Abbreviations

IgG4-RD: IgG4-related disease
CT: Contrast-enhanced computed tomography
MRI: Magnetic resonance imaging
αSMA: α-smooth muscle actin
ALK: Anaplastic lymphoma kinase
HPF: High-power fields
FDG-PET: ^18^ F-fluorodeoxyglucose-positron emission tomography
IMT: Inflammatory myofibroblastic tumor
